# Aspirin down Regulates Hepcidin by Inhibiting NF-κB and IL6/JAK2/STAT3 Pathways in BV-2 Microglial Cells Treated with Lipopolysaccharide

**DOI:** 10.3390/ijms17121921

**Published:** 2016-12-16

**Authors:** Wan-Ying Li, Fei-Mi Li, Yu-Fu Zhou, Zhong-Min Wen, Juan Ma, Ke Ya, Zhong-Ming Qian

**Affiliations:** 1 Laboratory of Neuropharmacology, Fudan University School of Pharmacy, Shanghai 201203, China; lj19840208@126.com (W.-Y.L.); lfm19910916@sina.com (F.-M.L.); zyf19850722@126.com (Y.-F.Z.); mj19890412@126.com (J.M.); 2 Department of Neurology, The Second Affiliated Hospital of Soochow University, Suzhou 215004, China; 3 School of Biomedical Sciences, Faculty of Medicine, The Chinese University of Hong Kong, Shatin, NT, Hong Kong, China

**Keywords:** aspirin, hepcidin, P65 (nuclear factor-κB), IL-6/JAK2/STAT3 pathway, lipopolysaccharide (LPS), nitric oxide (NO), iron regulatory protein 1 (IRP1)

## Abstract

Aspirin down regulates transferrin receptor 1 (TfR1) and up regulates ferroportin 1 (Fpn1) and ferritin expression in BV-2 microglial cells treated without lipopolysaccharides (LPS), as well as down regulates hepcidin and interleukin 6 (IL-6) in cells treated with LPS. However, the relevant mechanisms are unknown. Here, we investigate the effects of aspirin on expression of hepcidin and iron regulatory protein 1 (IRP1), phosphorylation of Janus kinase 2 (JAK2), signal transducer and activator of transcription 3 (STAT3) and P65 (nuclear factor-κB), and the production of nitric oxide (NO) in BV-2 microglial cells treated with and without LPS. We demonstrated that aspirin inhibited hepcidin mRNA as well as NO production in cells treated with LPS, but not in cells without LPS, suppresses IL-6, JAK2, STAT3, and P65 (nuclear factor-κB) phosphorylation and has no effect on IRP1 in cells treated with or without LPS. These findings provide evidence that aspirin down regulates hepcidin by inhibiting IL6/JAK2/STAT3 and P65 (nuclear factor-κB) pathways in the cells under inflammatory conditions, and imply that an aspirin-induced reduction in TfR1 and an increase in ferritin are not associated with IRP1 and NO.

## 1. Introduction

Aspirin is a non-steroidal anti-inflammatory drug (NSAID) and has been used for many years to treat a wide range of maladies, including pain and inflammation [1]. Preclinical and clinical studies have evidenced that aspirin has beneficial effects on mood disorders and schizophrenia and that high-dose aspirin is associated with a reduced risk of Alzheimer’s disease (AD) [2]. This oldest agent in medicine has also been considered to be a potential new therapy for a range of neuropsychiatric disorders [2].

Studies have demonstrated that aspirin and sodium salicylate have a significant neuro-protective role in 1-methyl-4-phenyl-1,2,3,6-tetrahydropyridine (MPTP) [3,4], rotenone [5], 1-methyl-4-phenylpyridiniumion (MPP^+^), and 6-hydroxydopamine (6-OHDA) [6] animal models in vivo and in neurons exposed to 6-OHDA and MPP^+^ in vitro [7]. MPTP, rotenone, 6-OHDA, and MPP^+^ are all neurotoxins known to trigger oxidative stress [4,7]. The neuro-protective effects of aspirin against oxidative stress induced by these neurotoxins have therefore been considered to be related to its ability to scavenger free radicals [3,4,6].

Abnormally high levels of iron and oxidative stress have been observed in a number of neurodegenerative disorders [8,9,10,11]. Iron is a major generator of reactive oxygen species (ROS), and oxidative stress resulting from increased iron in the brain has been widely considered to be an initial causes of neuronal death in some neurodegenerative diseases [12,13]. In addition, it has been demonstrated that aspirin can affect iron metabolism by increasing ferritin synthesis in the cultured bovine pulmonary artery endothelial cells [14] and reducing serum ferritin (SF) in humans [15].

The well-established association of inflammatory and expression of iron regulatory hormone hepcidin [16,17], the anti-inflammatory character of aspirin [2], and the findings as discussed above prompted us to speculate that aspirin might have the ability to affect iron metabolism. Recently, we therefore investigated the effects of aspirin on the expression of three major iron metabolism proteins, transferrin receptor 1 (TfR1), ferroportin 1 (Fpn1), and ferritin, as well as hepcidin and interleukin 6 (IL-6) in BV-2 microglial cells. We found that aspirin significantly down regulates TfR1 and up regulates Fpn1 and ferritin expressions in cells treated without lipopolysaccharides (LPS) in vitro, as well as down regulates hepcidin and IL-6 levels in cells treated with LPS [18]. However, the relevant mechanisms are unknown. In the present study, we investigate the effects of aspirin on expression of hepcidin mRNA and regulating molecules of hepcidin, including IL-6 mRNA, iron regulatory protein 1 (IRP1) protein, phosphorylation of Janus kinase 2 (JAK2), signal transducer and activator of transcription 3 (STAT3), P65 (nuclear factor-κB, NF-κB), and nitric oxide (NO) in BV-2 microglial cells treated with and without LPS.

## 2. Results

### 2.1. Aspirin Protects BV-2 Microglial Cells from Lipopolysaccharides (LPS)-Induced Damage

**Figure 1:**
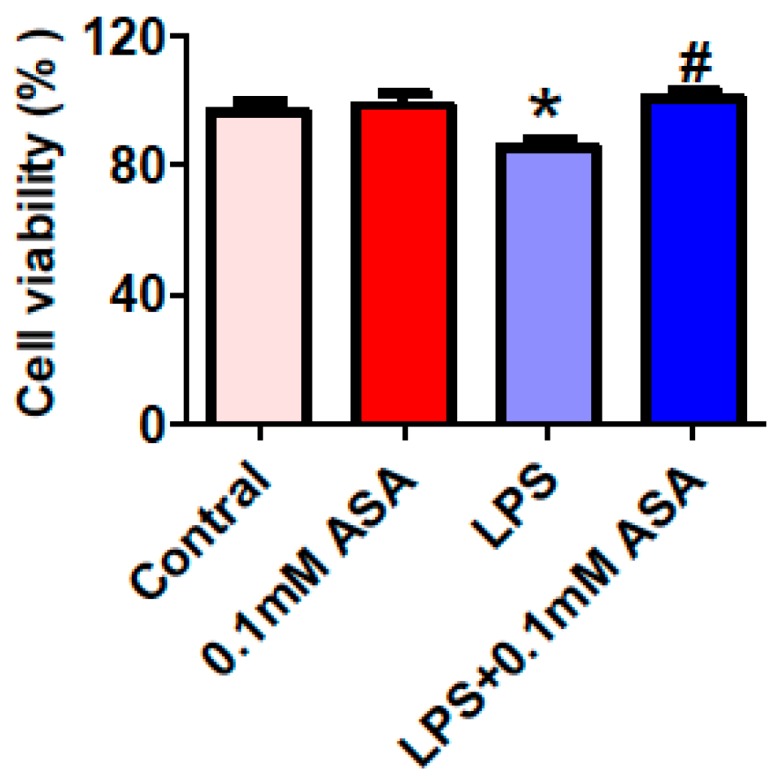
Aspirin protects BV-2 microglial cells from LPS-induced damage. BV-2 microglial cells were treated with 0.1% ethanol (Control) or aspirin (ASA) for 24 h—0.1% ethanol for 18 h and then 1 µg/mL of LPS for another 6 h (LPS) or aspirin for 18 h and then 1 µg/mL of LPS for another 6 h (LPS + 0.1 mM ASA). Cell viability was then conducted as described in Materials and Methods. Data were represented as means ± SEM (*n* = 4). * *p* < 0.05 vs. the control; # *p* < 0.05 vs. the LPS-treated group.

We first investigated the effects of aspirin (ASA) on the cell viability by treating BV-2 microglial cells with a vehicle (0.1% ethanol) for 24 h (The Control), 0.1 mM aspirin for 24 h (ASA), 0.1% ethanol for 18 h + 1 µg/mL of LPS for 6 h (LPS), or aspirin for 18 h + 1 µg/mL of LPS for 6 h (LPS + 0.1 mM ASA). We used 0.1 mM aspirin because this concentration was found to have a significant effect on hepcidin mRNA expression in LPS-treated BV-2 microglial cells in another recent study [18]. The 3-(4,5-dimethylthiazol-2-yl)-2,5-diphenyltetrazolium bromide (MTT) assay showed that there was no significant difference in cell viability between cells treated with the vehicle (control) or with 0.1 mM aspirin (Figure 1). It was also found that the cell viability in cells treated with LPS alone were significantly lower than those in the cells treated with the vehicle or the 0.1 mM aspirin, implying that LPS could induce cell-damage under our in vitro experimental conditions. However, the viability of the cells treated with 0.1 mM aspirin plus LPS was significantly higher than that of the cells treated with LPS alone and almost the same as that of control cells (Figure 1). This result is consistent with our recent findings [18] and re-confirmed that aspirin has a role in protecting BV-2 microglial cells from LPS-induced damage in vitro.

### 2.2. Aspirin Inhibits Hepcidin mRNA Expression But Has No Effect on IRP1 Protein Expression in BV-2 Microglial Cells Treated with LPS

**Figure 2:**
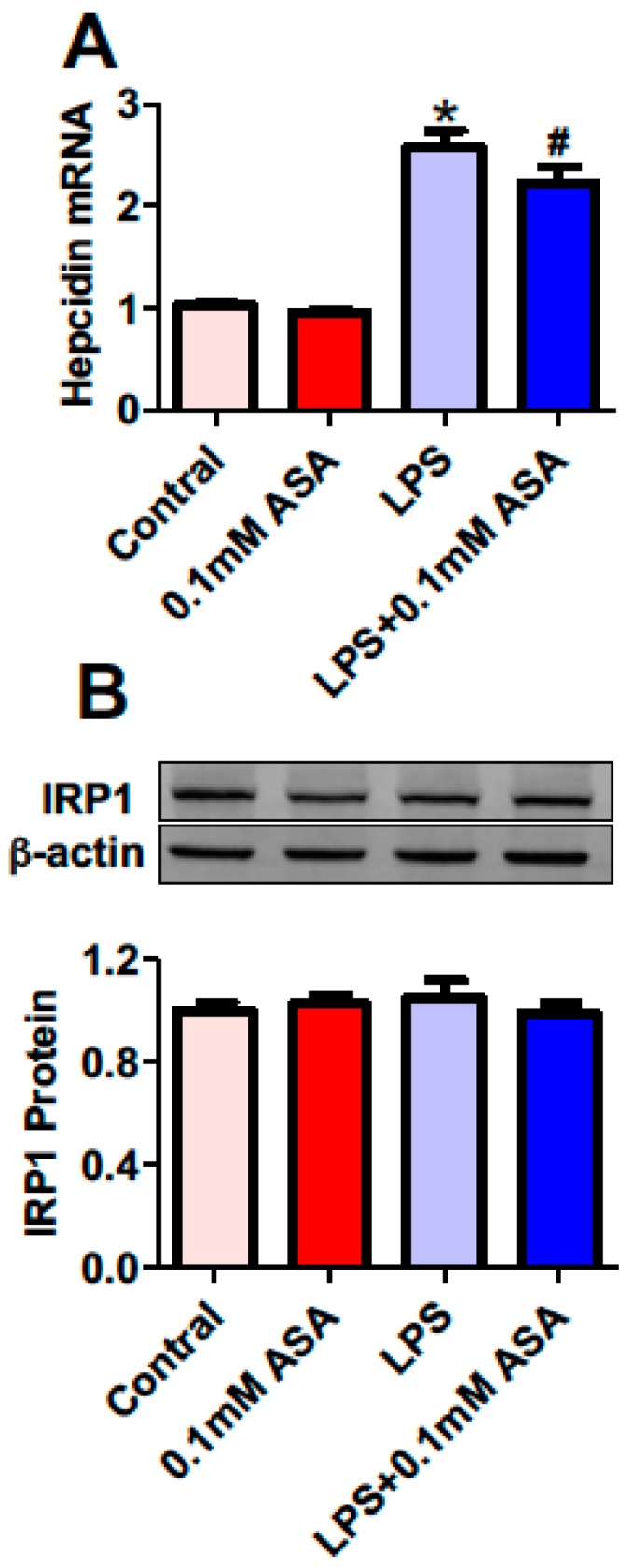
Aspirin inhibits hepcidin mRNA expression but has no effect on IRP1 protein expression in BV-2 microglial cells treated with LPS. BV-2 microglial cells were treated with 0.1% ethanol (Control) or aspirin (ASA) for 24 h—0.1% ethanol for 18 h and then 1 µg/mL of LPS for another 6 h (LPS) or aspirin for 18 h and then 1 µg/mL of LPS for another 6 h (LPS + 0.1 mM ASA). Expression of hepcidin mRNA (**A**) and IRP1 protein (**B**) were measured by RT-PCR and Western blot analysis, respectively. Data were presented as mean ± SEM (*n* = 5). * *p* < 0.05 vs. the control; # *p* < 0.05 vs. the LPS-treated group.

We then investigated the effects of aspirin on hepcidin mRNA and IRP1 protein expression in BV-2 microglial cells by treating with a vehicle (0.1% ethanol) for 24 h (The Control), 0.1 mM aspirin for 24 h (ASA), 0.1% ethanol for 18 h + 1 µg/mL of LPS for 6 h (LPS), or aspirin for 18 h + 1 µg/mL of LPS for 6 h (LPS + 0.1 mM ASA). Treatment with LPS induced a significant increase in hepcidin mRNA expression, the levels of hepcidin mRNA in the cells treated with LPS being markedly higher than those in the controls (Figure 2A). However, hepcidin mRNA expression in the cells treated with aspirin plus LPS was significantly lower than that in the cells treated with LPS only, but there was no difference between the cells treated with aspirin or with the vehicle. These results demonstrated that aspirin could inhibit hepcidin mRNA expression in BV-2 microglial cells treated with LPS but not in the cells treated without LPS. Western blot analysis showed that there were no differences in IRP1 protein content between cells treated with aspirin or the vehicle, or with LPS or aspirin plus LPS (Figure 2B), evidencing that aspirin has no effect on IRP1 protein expression in BV-2 microglial cells treated with or without LPS.

### 2.3. Aspirin Inhibits Phosphorylation of JAK2, STAT3, and P65(NF-κB) and Expression of IL-6 mRNA in BV-2 Microglial Cells Treated with or without LPS

**Figure 3:**
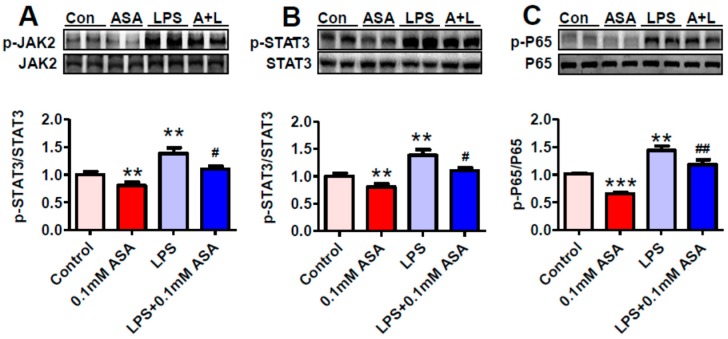
Aspirin inhibits phosphorylation of JAK2, STAT3, and P65(NF-κB) in BV-2 microglial cells treated with or without LPS. BV-2 microglial cells were treated with 0.1% ethanol (Control) or aspirin (ASA) for 24 h—0.1% ethanol for 18 h and then 1 µg/mL of LPS for another 6 h (LPS) or aspirin for 18 h and then 1 µg/mL of LPS for another 6 h (LPS + 0.1 mM ASA). Phosphorylation of JAK2 (**A**); STAT3 (**B**); and P65(NF-κB) (**C**) was detected by Western blot analysis, as described in Materials and Methods. Data were represented as mean ± SEM (*n* = 5). ** *p* < 0.01; *** *p* < 0.001 vs. the control; # *p* < 0.05; ## *p* < 0.01 vs. the LPS-treated group.

**Figure 4:**
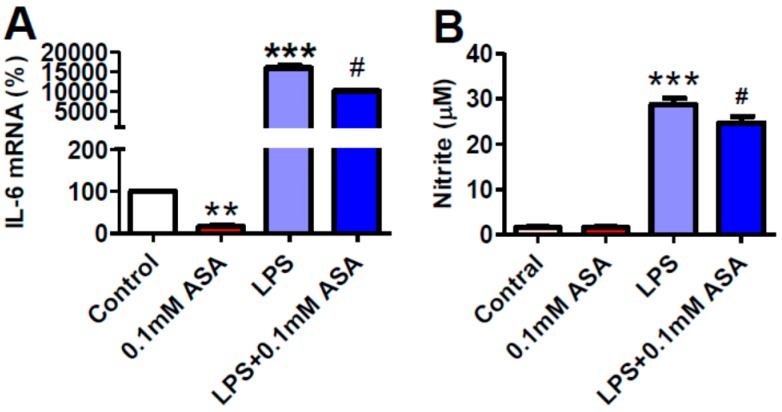
Aspirin inhibits expression of IL-6 mRNA in BV-2 microglial cells treated with or without LPS and NO production in BV-2 microglial cells treated with LPS but not in the cell treated without LPS. BV-2 microglial cells were treated with 0.1% ethanol (Control) or aspirin (ASA) for 24 h—0.1% ethanol for 18 h and then 1 µg/mL of LPS for another 6 h (LPS) or aspirin for 18 h and then 1 µg/mL of LPS for another 6 h (LPS + 0.1 mM ASA). Expression of IL-6 mRNA (**A**) was measured by RT-PCR and production of NO (**B**) was assayed by measuring the levels of nitrite (a metabolite of NO) in culture medium as described in Materials and Methods. Data were represented as mean ± SEM (*n* = 5). ** *p* < 0.01; *** *p* < 0.001 vs. the control; # *p* < 0.05 vs. the LPS-treated group.

To understand why aspirin could inhibit hepcidin expression under inflammatory conditions, we investigated the effects of aspirin on phosphorylation of JAK2, STAT3, and P65 by incubating BV-2 microglial cells with a vehicle (0.1% ethanol) for 24 h (The Control), 0.1 mM aspirin for 24 h (ASA), 0.1% ethanol for 18 h + 1 µg/mL of LPS for 6 h (LPS), or aspirin for 18 h + 1 µg/mL of LPS for 6 h (LPS + 0.1 mM ASA). It was found that the contents of p-JAK2 (Figure 3A), p-STAT3 (Figure 3B), p-P65 (Figure 3C), and IL-6 mRNA (Figure 4A) in the cells treated with LPS were significantly higher than those in the control cells as well as in the cells treated with aspirin plus LPS. This implied that LPS could dramatically increase JAK2, STAT3, and P65(NF-κB) phosphorylation and IL-6 mRNA expression, while aspirin was able to attenuate the LPS-induced increase in phosphorylation and expression. In addition, our findings showed that the levels of IL-6, p-JAK2, p-STAT3, and p-P65(NF-κB) in the cells treated with aspirin only were significantly lower than those in the control cells, suggesting that aspirin was able to inhibit IL-6 mRNA expression, JAK2, STAT3, and P65(NF-κB) phosphorylation under ‘normal’ conditions in vitro, not only under inflammatory conditions in vitro.

### 2.4. Aspirin Inhibits NO Production in BV-2 Microglial Cells Treated with LPS But Not in the Cell Treated without LPS

In a previous study, we demonstrated that aspirin down regulates TfR1 and up regulates Fpn1 and ferritin expression in BV-2 microglial cells in vitro; however, the mechanisms are unknown. It has been documented that NO can regulate the expression of TfR1 and ferritin by interacting with IRP1 [19,20,21]. To find out whether NO is involved in the effects of ASA on TfR1 and ferritin expression, we also investigated the effects of ASA on NO production in the cells treated with or without LPS. Treatment with LPS was found to induce a significant increase in NO levels, while pre-treatment with ASA displayed a marked inhibition on the LPS-induced increase in NO production, the levels of NO being significantly lower in ASA + LPS-treated cells than in LPS-treated cells (Figure 4B). There were no differences in NO content between the ASA-treated and the control cells.

## 3. Discussion

It has been well-demonstrated that LPS is able to up regulate hepcidin expression [22] via the IL-6/STAT3 signaling pathway and then down regulate expression of TfR1 and Fpn1 in the brain [23]. In our recent study, we [18] demonstrated that aspirin significantly inhibits the LPS-induced increase in IL-6 and hepcidin mRNA expression and revises the LPS-evoked reduction in TfR1 and Fpn1 expression in BV-2 microglial cells. The inhibition of aspirin on IL-6 and hepcidin mRNA expression suggests that aspirin might have play a role in suppressing the activated IL-6/ JAK2/STAT3 signaling pathway in LPS-treated BV-2 microglial cells. To test this hypothesis, we investigated the effects of aspirin on the contents of IL-6 mRNA, p-JAK2, and p-STAT3 in LPS-treated BV-2 microglial cells. We demonstrated that the significant increase in expression of IL-6 mRNA as well as phosphorylation of JAK2 and STAT3 induced by LPS could be largely suppressed by pre-incubation of the cells with aspirin. These findings provide evidence that aspirin down regulates hepcidin at least partly by inhibiting the IL6/JAK2/STAT3 pathway and then alleviates the LPS-induced reduction in TfR1 and Fpn1 expression in LPS-treated BV-2 microglial cells.

The transcription factor NF-κB is critical for the inducible expression of multiple cellular and viral genes involved in inflammation and infection including IL-6 [24,25]. Studies have demonstrated that aspirin and its metabolite sodium salicylate (another anti-inflammatory drug) are both able to inhibit the activation of NF-κB by inhibiting the activity of IκB kinase-beta (IκB-β) to preventing the translocation of NF-κB to the nucleus [24,26,27]. Evidence also shows that the toll-like receptor 4 (TLR4) recognition of LPS (TLR4 ligand), a pathogen-associated molecular pattern, results in the triggering of downstream signaling cascades leading to the activation of NF-κB [25]. These led us to speculate that the activation of NF-κB might play a role in the LPS-induced increase in hepcidin mRNA expression, while the inhibition of aspirin on hepcidin mRNA expression might be partly associated with its role in inhibiting the activation of NF-κB, as has been found in human peripheral blood leukocytes [28]. We therefore investigated the effects of aspirin on P65(NF-κB) phosphorylation in BV-2 microglial cells treated with LPS. P65(NF-κB) was examined here because the RelA(p65)–p50 heterodimer is the most frequently activated form of NF-κB in TLR signaling [29]. Our findings show that LPS can induce a marked increase in phosphorylation of P65(NF-κB), which can be significantly suppressed by the pre-incubation of cells with aspirin. This implies that the down regulation of hepcidin might also be partly associated with the inhibiting role of aspirin on NF-κB phosphorylation in LPS-treated BV-2 microglial cells.

It was noticed that aspirin was able to inhibit levels of phosphorylation of JAK2, STAT3, and P65(NF-κB) not only under in vitro inflammatory conditions but also in vitro ‘normal’ conditions. The levels of p-JAK2, p-STAT3, and p-P65 were found to be significantly lower in cells treated with ASA than those in the control cells. In theory, this could lead to a reduction in hepcidin mRNA in cells treated with ASA only. However, no difference was found in the contents of hepcidin mRNA between the cells treated with ASA and control cells. Under in vitro ‘normal’ conditions, the base-line level of JAK2, STAT3, and P65 is relatively lower, as compared with in vitro inflammatory conditions. Although aspirin can induce a reduction in the content of these mediators, the reduction might not be enough to result in significant changes in hepcidin mRNA expression. This is probably one of causes for the inconformity in the response of mediators and hepcidin to aspirin. Further studies on this possibility and other relevant causes are needed.

In a previous study, we demonstrated that aspirin down regulates TfR1 and up regulates Fpn1 and ferritin expression in BV-2 microglial cells treated without LPS [16]; however, the mechanisms are unknown. The responses of TfR1, Fpn1, and ferritin to aspirin are absolutely unrelated to hepcidin because the peptide has no response to aspirin under in vitro “normal” conditions. Like hepcidin, IRP1 is a key protein involved in the regulation of iron homeostasis. In most types of cells, the coordinated control of TfR1 and ferritin by cellular iron is mediated by IRP1 [30,31]. In addition, it has been documented that NO regulates expression of TfR1 and ferritin by interacting with IRP1 [19,20,21]. To find out why aspirin is able to down regulate TfR1 and up regulate Fpn1 and ferritin expression in BV-2 microglial cells under in vitro “normal” conditions, we examined the effects of aspirin on IRP1 expression and NO production in BV-2 microglial cells. We found that the levels of IRP1 as well as the NO in the cells treated with aspirin are no significantly different from those in the control cells. This finding implies that an aspirin-induced reduction in TfR1 and an increase in ferritin are not associated with IRP1 and NO and suggest that there might be some unknown mechanism by which aspirin regulates TfR1 and ferritin expression under in vitro “normal” conditions.

Aspirin inhibits inflammation mainly through its ability to suppress cyclooxygenase (COX) activity [32]. There are two isoforms of this enzyme—COX1 and COX2 [33]. COX-1 is constitutively expressed in most tissues and produces prostanoids responsible for normal physiological functions. COX-2 is sparsely present in most healthy tissues [33] and functions as a key enzyme for prostaglandin biosynthesis. In addition, COX-2 has been shown to contribute to the LPS-induced inflammatory process [34,35]. Cellular responses to inflammatory stimuli, including LPS, mainly involve the activation of mitogen-activated protein kinase (MAPK) signaling cascades [36,37,38]. The p38MAPK and JNK subfamilies play critical roles in regulating expression of pro-inflammatory mediators such as COX-2 and interleukins such as IL-6 [39,40,41,42]. These data imply that aspirin should be able to inhibit a LPS-induced increase in COX2 expression in BV-2 microglial cells, probably via inhibiting MAPK/JNK pathway, although the content of COX2 was not measured in this study. In addition, we found that the tendency in the effect of aspirin, LPS, or both on IL-6, JAK2, and P65 is very similar to that on COX2 reported by others [34]. This might suggest that COX2 may play a role in the down regulation of hepcidin induced by aspirin in LPS-treated BV-2 microglial cells. Further studies on this possibility are needed.

## 4. Materials and Methods

### 4.1. Chemicals

Unless otherwise stated, all chemicals, including aspirin (ASA), LPS (*Escherichia coli* 055:B5), MTT (3-(4,5-dimethylthiazol-2-yl)-2,5-diphenyltetrazolium bromide), and mouse monoclonal anti-β-actin, were obtained from Sigma Chemical Co., St. Louis, MO, USA. A BCA protein assay kit and a Revert Aid First Strand cDNA Synthesis Kit were purchased from Thermo Scientific, Waltham, MA, USA, and TRIZOL Reagent from Life technologies, Carlsbad, CA, USA. Rabbit monoclonal anti-phospho-JAK2, rabbit monoclonal anti-JAK2, rabbit polyclonal anti-phospho-STAT3, mouse monoclonal anti-STAT3, rabbit monoclonal anti-phospho-P65, and rabbit monoclonal anti-P65 antibodies were supplied by Cell Signaling Technology, Inc., Danvers, MA, USA, and mouse monoclonal anti-IRP1 was from Abcam, San Francisco, CA, USA. Goat anti-rabbit and anti-mouse IRDye 800CW secondary antibodies were bought from LI-COR bio sciences, Lincoln, NE, USA. The Health Department of Hong Kong and Shanghai Government and the Animal Research Ethics Committee of The Chinese University of Hong Kong (the project identification code: GRF14106914, 1 January 2015) and Fudan University (the project identification code: NSFC31271132, 1 January 2013; NSFC31330035, 1 January 2014) approved the experimental procedures of this study.

### 4.2. BV-2 Microglia Cells

BV-2 microglia cells (a murine microglia cell line) were grown in a 5% CO_2_ incubator at 37 °C in Dulbecco’s modified Eagle’s medium (DMEM) supplemented with 10% FBS (PAN Biotech, Aidenbach Bavaria, Germany) and antibiotics (penicillin 100 U/mL, streptomycin 100 mg/mL), and culture medium was changed every 2 days [24]. Then, BV-2 cells were seeded in 96-well plates (6 × 10^3^ cells/well) for cell viability assay, and 6-well plates (4 × 10^5^ cells/well) for RT-PCR and (8 × 10^5^ cells/well) for Western blotting analysis [18]. The cells were treated with different concentrations of 0.1 mM aspirin (in 0.1% ethanol) and/or LPS (1 mg/mL in PBS), which were dissolved in fresh DMEM without serum.

### 4.3. Assessment of Cell Viability

The cell viability were measured using an MTT assay as described previously [43]. Briefly, a total of 25 µL of MTT (1 g/L in PBS) was added to each well before the conduction of incubation at 37 °C for 4 h. The assay was stopped by the addition of a 100 µL of lysis buffer (20% SDS in 50% *N*′*N*-dimethylformamide, pH 4.7). Optical density (OD) was measured at the 570 nm wavelength by the use of an ELX-800 microplate assay reader (Bio-tek, Winooski, VT, USA), and the results were expressed as a percentage of absorbance measured in the control cells.

### 4.4. NO Production Assay

Production of NO was assayed by measuring the levels of nitrite (a metabolite of NO) in the culture medium using a colorimetric assay with Griess reagent according to Kim et al. [44], After 24 h of treatment with LPS with or without ASA, the culture media were collected and reacted with an equal volume of Griess reagent in 96-well culture plates and were incubated at room temperature for 10 min in the dark. The absorbance was measured at 540 nm using a microplate reader, and nitrite concentrations were calculated by reference to a standard curve generated by known concentrations of sodium nitrite.

### 4.5. Quantitative Real-Time PCR

Extraction of total RNA and preparation of cDNA were performed using a TRIZOL reagent andreverse transcription kit (Thermo Scientific) in accordance with the instruction of the manufacturers, respectively. The specific primers used for PCR are as follows: hepcidin forward, 5′-GAAGGCAAGATGGCACTAAGCA-3′; hepcidin reverse, 5′-TCTCGTCTGTTGCCGGAGATAG-3′; IL-6 forward, 5′-GAGGATACCACTCCCAACAGACC-3′; IL-6 reverse, 5′-AAGTGCATCATCGTTGTTCATACA-3′; β-actin forward, 5′-AAATCGTGCGTGACATCAAAGA-3′; β-actin reverse, 5′-GCCATCTCCTGCTCGAAGTC-3′ [19,45]. Quantitative real-time PCR was conducted with CFX96 PCR instrument (Bio-Rad, Hercules, CA, USA) using specific primers and SYBR Premix II kit (Takara, Dalian, China). The *C*_t_ values of each target gene were normalized to that of the β-actin mRNA. Relative gene expression was calculated by the 2^−ΔΔ*C*t^ method [46].

### 4.6. Western Blot Analysis

The cells were washed and lysed as described previously [47,48]. After centrifugation at 13,200× *g* for 15 min at 4 °C, the supernatant was collected, and protein content was determined using the BCA protein assay kit. Aliquots of the extract containing about 20 µg of protein were loaded and run on a single track of 10% SDS-PAGE under reducing conditions and subsequently transferred to a pure nitrocellulose membrane (Bio-Rad, Hercules, CA, USA). The blots were blocked and then incubated with primary antibodies—rabbit monoclonal anti-phospho-JAK2 (1:1000), rabbit monoclonal anti-JAK2 (1:1000), rabbit polyclonal anti-phospho-STAT3 (1:1000), mouse monoclonal anti-STAT3 (1:1000), rabbit monoclonal anti-phospho-P65 (1:1000), rabbit monoclonal anti-P65 (1:1000), and mouse monoclonal anti-IRP1 (1:1000) antibodies—overnight at 4 °C. After the incubation, the blots were washed three times and then incubated with goat anti-rabbit (1:1000) or anti-mouse IRDye 800 CW secondary antibodies (1:5000) for 2 h at room temperature. The intensity of the specific bands was detected and analyzed by the Odyssey infrared image system (Li-Cor, Lincoln, NE, USA). To ensure even loading of the samples, the same membrane was probed with a mouse monoclonal anti-β-actin antibody at a 1:5000 dilution.

### 4.7. Statistical Analysis

Statistical analyses were performed using Graphpad Prism. Data were presented as mean ± SEM. The differences between the means were all determined by a two-way analysis of variance (ANOVA). A probability value of *p* < 0.05 was considered statistically significant. 

## 5. Conclusions

We demonstrated that aspirin inhibits hepcidin mRNA as well as NO production in cells treated with LPS, but not in cells without LPS, suppresses IL-6, JAK2, STAT3, and P65(NF-κB) phosphorylation, and has no effect on IRP1 protein in cells treated with or without LPS. The findings provided evidence that aspirin down regulates hepcidin by inhibiting IL6/JAK2/STAT3 as well as P65(NF-κB) pathways in cells under inflammatory conditions and implies that an aspirin-induced reduction in TfR1 and an increase in ferritin were not associated with IRP1 and NO.

## Abbreviations

ASA	aspirin
Fpn1	ferroportin 1
IL-6	interleukin 6
IRP1	iron regulatory protein 1
JAK2	Janus kinase 2
LPS	lipopolysaccharides
NO	nitric oxide
NSAID	non-steroidal anti-inflammatory drug
P65(NF-κB)	rela (p65) nuclear factor-κB
SF	serum ferritin
STAT3	signal transducer and activator of transcription 3
TfR1	transferrin receptor 1
